# Effects of fluoride on proliferation and mineralization in periodontal ligament cells *in vitro*

**DOI:** 10.1590/1414-431X20165291

**Published:** 2016-07-11

**Authors:** K.Q. Li, S.S. Jia, M. Ma, H.Z. Shen, L. Xu, G.P. Liu, S.Y. Huang, D.S. Zhang

**Affiliations:** Department of Oral and Maxillofacial Surgery, Shandong Provincial Hospital Affiliated to Shandong University, Jinan, China

**Keywords:** Fluoride, Periodontitis, Periodontal ligament cells, Mineralization

## Abstract

Fluoride, which is often added to toothpaste or mouthwash in order to protect teeth from decay, may be a novel therapeutic approach for acceleration of periodontal regeneration. Therefore, we investigated the effects of fluoride on proliferation and mineralization in human periodontal ligament cells *in vitro*. The periodontal ligament cells were stimulated with various concentrations of NaF added into osteogenic inductive medium. Immunohistochemistry of cell identification, cell proliferation, alkaline phosphatase (ALP) activity assay, Alizarin red S staining and quantitative real-time-polymerase chain reaction (RT-PCR) were performed. Moderate concentrations of NaF (50-500 μmol/L) had pro-proliferation effects, while 500 μmol/L had the best effects. ALP activity and calcium content were significantly enhanced by 10 μmol/L NaF with osteogenic inductive medium. Quantitative RT-PCR data varied in genes as a result of different NaF concentrations and treatment periods. We conclude that moderate concentrations of NaF can stimulate proliferation and mineralization in periodontal ligament cells. These *in vitro* findings may provide a novel therapeutic approach for acceleration of periodontal regeneration by addition of suitable concentrations of NaF into the medication for periodontitis treatment, i.e., into periodontal packs and tissue patches.

## Introduction

Fluoride is found in teeth, bones, thyroid gland and skin of humans. Since reports of its strengthening effect on bones and teeth at recommended doses, it is officially considered to be a beneficial element for humans. However, chronic excessive intake of fluoride in water, diet and dentifrices may result in fluoride poisoning, which manifests as enamel fluorosis and bone injuries (1). Fluorosis extraperiosteal calcification and ossification could be detected during the development of severe skeletal fluorosis (2). It is generally accepted that fluoride-induced extraperiosteal ossification is closely associated with fibroblasts. Fibroblasts have been classified as inducible-osteogenic precursor cells that have an osteogenic function under special conditions (3). According to a previous study, the osteogenic function of fibroblasts induced by fluoride could play an important role in the development of extraperiosteal ossification during skeletal fluorosis (4). The authors suggest that, induced by excessive fluoride, fibroblasts could be stimulated to express osteogenic phenotype, increase osteogenic activity, and result in extraperiosteal ossification of bone injuries (2,4).

Periodontitis is a highly prevalent inflammatory oral disease. It can lead to the destruction of periodontal tissues and the loss of bone that surrounds the teeth. This disease is a major cause of adult tooth loss. Current treatments mainly aim at removing local stimulus and controlling inflammation. Afterwards, efforts are made to repair periodontal supporting tissues, which means achieving simultaneous regeneration of the lost alveolar bone, cementum and periodontal soft tissues. Unfortunately, in most cases, alveolar bone repair and regeneration following periodontitis treatment is still a challenge presented to dentists (5).

Periodontal tissues include alveolar bone, periodontal ligament, cementum and gums. The periodontal ligament is a specialized dense connective tissue embedded between the roots, cementum, and the alveolar bone. Early evidence shows that the periodontal ligament plays an important role in the maintenance of the periodontal tissue homeostasis by maintaining the balance between bone formation and resorption (6). It is composed of several different cell populations, such as fibroblasts, cementoblasts, osteoblasts, osteoclasts, and mesenchymal cells. Of all cells in the periodontal ligament, fibroblasts are the most numerous and of great importance in bone remodeling (7–9). Periodontal ligament fibroblasts are mesenchymal with intense contribution in periodontal ligament metabolism, collagen fibers synthesis, and metabolism of other constituent of the extracellular matrix (10). *In vitro* studies of cultured periodontal ligament fragments, periodontal ligament cells (PDLCs) are described as fiber-cord-like cells. They are reported as having multi-differentiation potential, being able to differentiate into fibroblasts, osteoblasts, and bone forming cells. Much of the research in recent years has confirmed that PDLCs can form bone nodules and express bone-related proteins, such as alkaline phosphatase (ALP), bone sialoprotein (BSP) and osteocalcin (OCN) under certain circumstances (11–14).

However, the majority of the studies investigating the effects of fluoride on osteogenic differentiation have used either osteoblasts or osteosarcoma cell lines, and currently no knowledge exists about the response of human PDLCs to fluoride. The primary objective of this study was to investigate the effects of fluoride on proliferation and mineralization in human PDLCs *in vitro*.

## Material and Methods

### Cell culture

PDLCs were obtained from healthy premolar or third molar teeth extracted from patients (11 to 23 years of age) for orthodontic treatment or preventive procedures. Teeth were placed in a minimum essential medium (ɑ-MEM; Hyclone, USA), containing 20% fetal bovine serum (FBS; Biological Industries, USA), 100 U/mL of penicillin, and 100 mg/mL of streptomycin (Gibco, USA). Teeth were then washed with sterile phosphate-buffered saline (PBS) containing 100 U/mL of penicillin, and 100 mg/mL of streptomycin as many times as possible until all blood and bone fragments were rinsed away. Single-use sterile scalpel was used to carefully scrap the periodontal ligament tissue from the middle third of the root surface. Tissue was centrifuged at 190 *g* at room temperature for 5 min. Digestion of collected tissue was done with 3 mg/mL of collagenase type I and 4 mg/mL of dispase (Sigma-Aldrich, USA) for 15 min with shaking every 5 min in constant temperature in 37°C water bath. After terminating digestion, centrifugation described as before was again performed. The supernatant was removed and the precipitate collected, pooled and plated into a 12.5-cm^2^ cell culture flask (Corning, USA) with 2 mL complete ɑ-MEM medium. The flask was placed vertically into a humidified incubator with 37°C constant temperature, 95% air, 5% CO_2_ for 4 h; it was then placed horizontally. Medium was replaced every 3 days until cell growth of 70-80% confluency. Digestion and passage was done with 0.25% trypsin protease (Gibco). Then cells were again incubated with complete ɑ-MEM medium with 10% FBS, 100 U/mL of penicillin, and 100 mg/mL of streptomycin. Cultures between passage 3 and 6 were used in the experiments.

### Immunohistochemistry

First, the cells were attached to cell slides at a density of 5×10^4^ cells per well in 24-well plates (Eppendorf, Germany) with complete culture medium. After 48 h, the cell slides were gently rinsed with PBS three times and fixed with 4% paraformaldehyde (PFA; Boster, China) for 20 min. Finally, the chromogenic reaction was performed with a diaminobenzidine kit (Beijing Zhongshan Golden Bridge Biotechnology, China). The cell slides were incubated with primary antibodies against vimentin and cytokeratins (Beijing Zhongshan Golden Bridge Biotechnology) at a dilution of 1:50 for 18 h at 4°C. At the same time, PBS was used as a substitute for the primary antibodies as a negative control.

### CCK-8 assay

Effects of various concentrations of NaF (Sigma, USA) on PDLCs proliferation was measured using a cell counting kit (CCK-8; Beyotime, China). PDLCs were seeded into 96-well culture plates (Eppendorf) with a concentration of 1×10^3^ cells/well. After 24-h incubation at 37°C with 5% CO_2_, the plates were treated with 0, 1, 5, 10, 50, 100, 5×10^2^, 1×10^3^, and 5×10^3^ μmol/L NaF for 1, 2, 3, 4, 5, and 6 days. CCK-8 was mixed with serum-free ɑ-MEM medium at a proportion of 1:10 in advance. After removal of complete ɑ-MEM medium, 110 μL mixture was added to each well and incubated at 37°C for 2 h, until the media turned yellow. Groups without cells were used as zero setting. We assessed cell viability by absorbance values in each well, which was measured with a spectrophotometer (Thermo, Finland) at a wavelength of 450 nm. Data were calculated using averages of three wells, and untreated PDLCs were considered as the control group. The concentrations of 0, 10, 5×10^2^ and 1×10^3^ μmol/L were chosen for subsequent experiments.

### NaF treatment

NaF was added to osteogenic inductive culture medium. There were four treatment groups: 1) osteogenic medium (basic culture medium supplemented with 10 mM/L beta-glycerophosphate, 0.1 μm/L dexamethasone, and 50 μg/L L-ascorbic acid; all from Sigma), 2) osteogenic medium supplemented with 10 μmol/L NaF, 3) osteogenic medium supplemented with 5×102 μmol/L NaF, and 4) osteogenic medium supplemented with 1×103 μmol/L NaF. The PDLCs were treated the same way in subsequent experiments. The medium was replaced every 3 days.

### ALP activity assay

PDLCs were plated at a density of 1×10^4^ cells/well in 24-well culture plates (Eppendorf) and cultured in basal medium. When cells reached 70-80% confluency, plates were treated with different concentrations of NaF. For ALP staining, cells triggered for 14 days were washed three times with PBS and fixed with 4% PFA for 20 min at room temperature. The fixed cells were stained with a BCIP/NBT ALP Color Development Kit (Leagene, China). Double-distilled water was added to each well to stop reaction after 30min. The BCIP/NBT method produced an insoluble blue NBT formazan. ALP stained areas were measured quantitatively with Image-Pro Plus 6.0 (Media Cybernetics, USA).

### Quantitative real-time polymerase chain reaction (RT-PCR)

After reaching 70-80% confluency from PDLCs seeded in 6-well plates at a density of 1×10^5^ cells/well, different concentrations of NaF were added as previously described for 7, 14, and 21 days. After treatments, the cells were lysed using RNAisoTM Plus (TakaRa, China) to isolate total RNAs. Single-stranded complementary DNA (cDNA) was obtained by reverse transcribing 1 μg of total RNA from each sample using PrimeScript^®^ RT reagent kit (TakaRa). Quantitative RT-PCR of Runt-related transcription factor 2 (Runx2), type I collagen α1 (COL1A1), ALP, OCN and glyceraldehyde 3-phosphate dehydrogenase (GAPDH) were performed on an equal amount of cDNA using SYBR^®^ Premix Ex TaqTM II (TakaRa) according to the manufacturer's instructions. Reactions were performed in triplicate on LightCycler^®^ 480 System Real-Time PCR (Roche, Switzerland). The cycling parameters used were: 95°C for 30 s, followed by 40 cycles of 95°C for 5 s, 60°C for 30 s, and 72°C for 30 s, and a dissociation program of 95°C for 15 s, 60°C for 30 s, and 95°C for 15 s. Melting curve analysis was included to assure that only one PCR product was formed. Expression of housekeeping gene GAPDH was amplified as an internal control. The relative amount of RNA was calculated by the 2^−ΔΔCt^ method. Primer and probe sequences for Runx2, COL1A1, ALP, and OCN, and GAPDH are shown in Table 1.

**Table:**
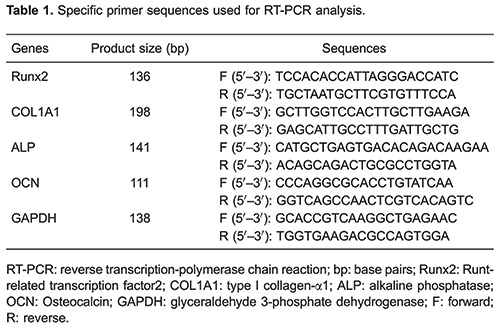

### Alizarin red S staining

PDLCs were subcultured in 24-well culture plates similarly to the ALP activity assay. Mineralization at 28 days was assessed by Alizarin red S staining, which stains the calcium minerals red. Cells were fixed in 4% PFA for 20 min and then stained with 2% Alizarin red S (pH 8.3; Leagene) solution with 500 µL/well for 30 min at room temperature. The excess dye was washed away with double-distilled water. After photographing, semi-quantitative measurement of mineralization was taken. The Alizarin red S-stained cultures were incubated with 500 µL 10% cetylpyridinium chloride (CTC; Meilun, China) for 15 min to solubilize and release calcium-bound Alizarin red into solution. The absorbance of the released Alizarin red S was measured at 562 nm in 96-well plates using a spectrophotometer. Groups of CTC were used as zero setting.

### Statistical analysis

Data are reported as mean±SD from at least three independent experiments. Data were analyzed by one-way analysis of variance (ANOVA) using SPSS software (SPSS Inc., USA). P values less than 0.05 were considered to be statistically significant.

## Results

### Cell culture and identification

**Figure 1:**
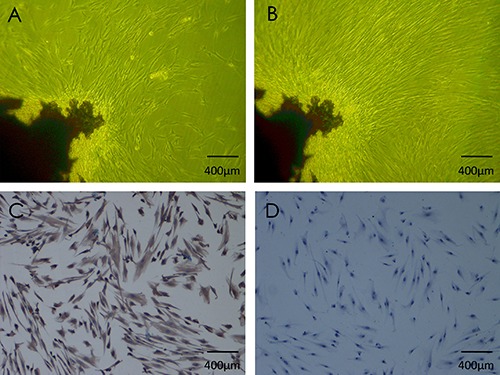
Periodontal ligament cell cultures and identification. *A*, primary cells shaped like fibroblasts and stretched radially from the tissue after 7-10 days. *B*, fiber-cord-like cells kept growing vigorously and reached 70-80% confluency after 10-14 days. *C*, immune histological staining was positive for vimentin. *D*, immune histological staining was negative for cytokeratin (all 100×).

In the course of primary cultured periodontal ligament cells, inverted microscope was used for observation of cell morphology and growth. After 7 to 10 days, adherent cells could be found radially stretching out from the edge of periodontal ligament fragments (Figure 1A). When approaching 70-80% confluency, fiber-cord-like cells kept growing vigorously (Figure 1B). Positive staining was observed for vimentin in the cytoplasm (Figure 1C), while staining was negative for cytokeratin (Figure 1D). Immunohistochemistry indicated that the cultured cells originated from mesenchyme and not from epithelium, information that can be used reliably in further experiments.

### Dose effects of fluoride on cell proliferation

**Figure 2:**
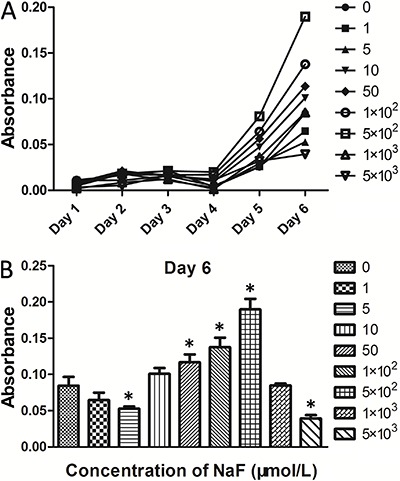
Effects of various concentrations of NaF (μmol/L) on cell proliferation of periodontal ligament cells. *A*, cell proliferation from days 1 to 6. *B*, cell proliferation at day 6. Data are reported as means±SD. *P<0.05 compared to the control group (ANOVA).

To study the effect of the various concentrations of NaF on PDLCs proliferation, we determined cell viability by CCK-8 assay after 1, 2, 3, 4, 5 and 6 days of culture. As shown in Figure 2A, there were no significant differences between the NaF concentrations and the control at days 1, 2 and 3 (P>0.05). On the contrary, from day 5 onwards, absorbance was significantly increased by the 50, 100, 5×10^2^ μmol/L treatments compared to the control, while 5×10^2^ μmol/L NaF caused the highest value at day 6 as shown in Figure 2B. However, when the concentration increased up to 5×10^3^ μmol/L, the absorbance exhibited a significant decrease compared to the control group (P<0.05), indicating a cytotoxic effect. These data indicate that NaF has dose-dependent effects on cell proliferation.

### ALP activity assay

**Figure 3:**
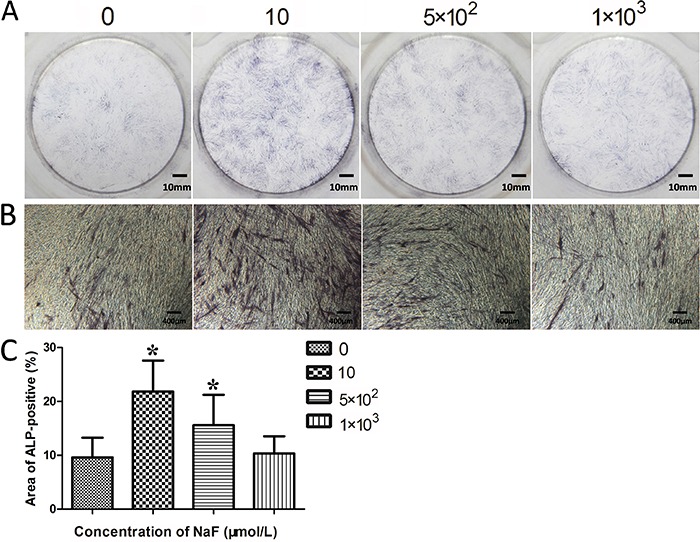
Effects of various concentrations of NaF (μmol/L) on the alkaline phosphatase (ALP) activity of periodontal ligament cells. *A*, ALP staining after 14 days. *B*, ALP staining after 14 days (100×). *C*, ALP positive areas collected by Image-Pro Plus 6.0 on day 14. Data are reported as means±SD. *P<0.05 compared to the control group (ANOVA).

As demonstrated in Figure 3, the ALP activity at day 14 was significantly promoted by the 10 and 5×10^2^ μmol/L treatment compared to the control, while 10 μmol/L NaF caused the highest ALP positive area (P<0.05). However, no significant differences were found between 1×10^3^ μmol/L-NaF-treated and untreated groups at the same time point (P>0.05). This suggests that supplementing with a moderate concentration of NaF could induce further mineralization of PDLCs in the presence of osteogenic inducers.

### Quantitative real-time polymerase chain reaction (RT-PCR)

**Figure 4:**
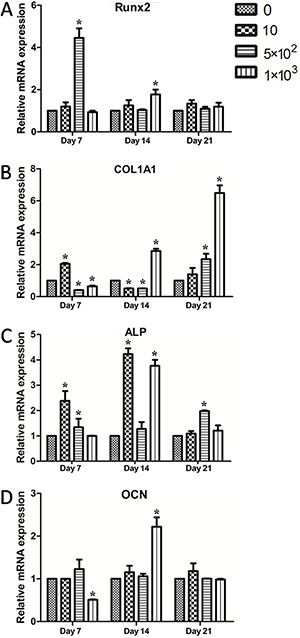
Effects of various concentrations of NaF (μmol/L) on the osteogenic differentiation markers of periodontal ligament cells with quantitative RT-PCR analysis. The results are standardized to the reference gene GAPDH, and reported as relative mRNA levels. *A*, Runx2 mRNA. *B*, type I collagen α1 (COL1A1) mRNA. *C*, alkaline phosphatase (ALP) mRNA. *D*, Osteocalcin (OCN) mRNA. Data are reported as means±SD. *P<0.05 compared to the control group (ANOVA).

The gene expression of osteogenic marker genes Runx2, COL1A1, ALP, OCN and GAPDH was studied at time points 7, 14 and 21 days for the purpose of further analysis of the osteogenic differentiation of PDLCs in response to the NaF treatment. As seen in Figure 4A, 5×10^2^ μmol/L NaF significantly increased expression of Runx2 at 7 days. In Figure 4B, the mRNA expression level of COL1A1 showed a significantly downward trend along with 5×10^2^ μmol/L NaF added for the first 7 and 14 days (P<0.05). However, as the culture time increased, COL1A1 mRNA expression was significantly increased on day 21 (P<0.05). ALP expression also showed the same change trend following 5×10^2^ μmol/L NaF addition (Figure 4C). Moreover, 10 μmol/L NaF increased ALP mRNA level at both 7 and 14 days (P<0.05). The increase of OCN mRNA expression level occurred at day 14 with 1×10^3^ μmol/L NaF (P<0.05) (Figure 4D). These data indicated that the exposure of fluoride for a certain period may influence cell differentiation.

### Alizarin red S staining

**Figure 5:**
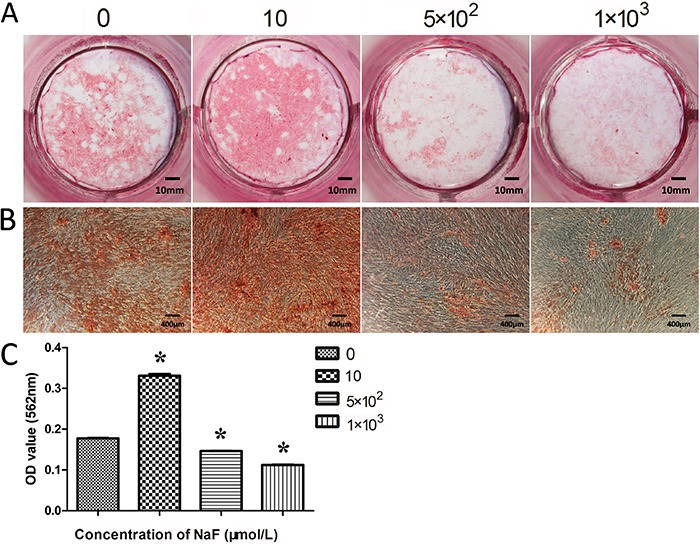
Effects of various concentrations of NaF on the calcified nodules formation of periodontal ligament cells. *A*, cultured PDLCs formed calcified nodules that stained positively for Alizarin red S staining after 28 days. *B*, Alizarin red S staining after 28 days (100×). *C*, Quantitative comparison of mineralized nodule formation among the groups. Data are reported as means±SD. *P<0.05 compared to the control group (ANOVA).

After 28 days of culture, the calcium content in the cultures was measured. The calcium content in the 10 μmol/L-NaF-treated group was higher than the control group (P>0.05). However, in the 5×10^2^ and 1×10^3^ μmol/L groups there was a significant reduction of the calcium content (P<0.05; Figure 5). This implied that mineralized matrix deposition gradually increased with 10 μmol/L NaF in the presence of osteogenic inducers. The addition of 5×10^2^ and 1×10^3^ μmol/L NaF resulted in reduced calcium deposition at day 28.

## Discussion

Much work has been done to study the dual effect of fluoride on bone formation. Although chronic excessive fluoride intake can result in skeletal fluorosis, low concentrations of fluoride are normally used to reduce the incidence of caries. In recent years, several investigations have suggested that fluoride could promote osteoblastic differentiation (4,15–18). PDLCs play an important role not only in the maintenance of periodontal ligament but also in the promotion of periodontal regeneration (19 [Bibr B20]). Previous reports showed that PDLCs have great potential for osteogenic differentiation (11–14,). However, effects of fluoride on proliferation and mineralization in human PDLCs *in vitro* have not been investigated. In this study, various concentrations of NaF were applied to PDLCs cultured in osteoinductive medium, and the results showed that fluoride could stimulate proliferation and mineralization *in vitro*.

The CCK-8 assay is a convenient and efficient approach for assessment of cell viability, which was used to measure the effects of NaF on proliferation in PDLCs. Our study indicates that NaF has dose-dependent effects on cell proliferation. Cell viability increased from 50 μmol/L concentration of NaF and reached a peak at 5×10^2^ μmol/L. At a higher NaF concentration of 5×10^3^ μmol/L, the cell viability declined compared with control group. Our results are similar to those of a human osteosarcoma MG-63 cells *in vitro* investigation, which demonstrated that a moderate concentration up to 5×10^2^ μmol/L of NaF has a pro-proliferation effect, while 2×10^3^ μmol/L NaF inhibited cell proliferation (15). According to another study examining the cellular mechanisms underlying the cytotoxicity induced by NaF, concentrations higher than 5×10^3^ μmol/L reduced the cell viability of human gingival fibroblasts (21). In a research of age-related changes of normal human oral cells sensitivity to NaF, NaF dose-dependently reduced the viable cell number. However, no beneficial (growth promoting) effect (the so-called "hormesis") was seen in any of the three normal human oral cell types (pulp cell, gingival fibroblast, periodontal ligament fibroblast), except for those cells at the terminal phase, indicating that cells became resistant to cytotoxicity induced by NaF with aging (22). We may confirm that the effect of NaF on cell proliferation is concentration-, time- and cell-type-dependent.

As described before, after supplementation with moderate concentrations of fluoride, a growth of PDLCs was stimulated. However, in order to identify a suitable concentration of NaF for novel therapeutic approaches for acceleration of periodontal regeneration, our experiment tested fluoride concentrations of 10, 5×10^2^ and 1×10^3^ μmol/L (low, medium, and high-dose NaF) on the premise that there was no cytotoxicity on PDLCs during the pilot period.

As known, ALP is an early well-defined marker of osteogenic differentiation, and higher ALP activity represents a greater potential for bone formation. In this investigation, we assessed effects of various concentrations of NaF on ALP activity of PDLCs. Previous studies indicate that the addition of fluoride could affect ALP activity in other cells with a potential for bone forming. Human bone marrow mesenchymal stem cells grown on fluoride-modified titanium implant specimens showed higher ALP activity compared with grit-blasted ones (23). In primary rat osteoblasts, concentrations from 0.1 up to 100 μmol/L NaF can significantly promote ALP activity compared with an untreated group (16). In agreement with that report, the present results showed that cells treated with 10 and 5×10^2^ μmol/L NaF showed a significant increase in ALP activity.

This study investigated the widely used bone formation markers Runx2, ALP, COL1A1 and OCN by RT-PCR against the housekeeping gene, GAPDH (16–18). PDLCs under differentiation could synthesize different products in different stages. Runx2 is an important transcription factor that plays a central role in bone formation (24). The expression of COL1A1, as well as of ALP, represents early osteogenic differentiation. Transcription of COL1A1 and OCN can be directly stimulated by Runx2 (25,26). In the present study, 5×10^2^ μmol/L NaF increased expression of Runx2 as well as proliferation. As the culture time increased, 1×10^3^ μmol/L NaF promoted COL1A1 mRNA expression. 10 μmol/L NaF only increased COL1A1 expression at 7 days. Similar to the ALP activity assessment, 10 μmol/L NaF increased ALP mRNA level at both 7 and 14 days. However, no significant difference in COL1A1 and ALP mRNA expression with 10 μmol/L NaF at 21 days was observed. PDLCs at this age might have already finished the conversion phrase, proliferation phase and cell aggregation secretory phase. OCN is a specific maker for late stages of osteoblastic differentiation, implying that at this time the cultured cells would present the phenotype of mature osteoblasts (27). Unlike other genes, the expression of OCN mRNA increased slightly in the 10 μmol/L NaF groups at 14 and 21 days, but these data had no statistical significance. Similar results were reported in fluoride-treated fibroblast and osteoblast at 48 h with no statistical significance in expression of OCN mRNA (4).

To further confirm the bone formation induced by fluoride in osteogenic medium, Alizarin red S staining was used to analyze mineralization in PDLCs *in vitro*. The result shows that 10 μmol/L NaF lead to an increased activity compared with the control group. In contrast, and to our surprise, both 5×10^2^ and 1×10^3^ μmol/L NaF presented an effect of inhibition on the calcified nodules formation of PDLCs at 28 days, since cells did not reduce the ALP activity. The exact reason for this finding was unclear; however, it might be explained by the different cell passages and the experimental conditions.

In conclusion, the results suggest that moderate concentrations of NaF can stimulate proliferation and mineralization in PDLCs. This information may provide a novel therapeutic approach for acceleration of periodontal regeneration by addition of a suitable concentration of NaF into the medication for periodontitis treatment as for instance, into periodontal packs and tissue patches.
